# Disrupted topological organization of white matter structural networks in high myopia patients revealed by diffusion kurtosis imaging and tractography

**DOI:** 10.3389/fnins.2023.1158928

**Published:** 2023-06-22

**Authors:** Huihui Wang, Hongwei Wen, Jing Li, Qian Chen, Shanshan Li, Zhenchang Wang

**Affiliations:** ^1^Department of Radiology, Beijing Chaoyang Hospital, Capital Medical University, Beijing, China; ^2^Department of Radiology, Beijing Friendship Hospital, Capital Medical University, Beijing, China; ^3^Key Laboratory of Cognition and Personality (Ministry of Education), Faculty of Psychology, Southwest University, Chongqing, China; ^4^Department of Ophthalmology, Beijing Friendship Hospital, Capital Medical University, Beijing, China

**Keywords:** high myopia, diffusion kurtosis imaging, white matter, structural network, graph theory analysis

## Abstract

**Introduction:**

High myopia (HM) is a public health issue that can lead to severe visual impairment. Previous studies have exhibited widespread white matter (WM) integrity damage in HM patients. However, how these WM damages are topologically related, and the network-level structural disruptions underlying HM has not been fully defined. We aimed to assess the alterations of brain WM structural networks in HM patients using diffusion kurtosis imaging (DKI) and tractography in the present study.

**Methods:**

Individual whole-brain and ROI-level WM networks were constructed using DKI tractography in 30 HM patients and 33 healthy controls. Graph theory analysis was then applied to explore the altered global and regional network topological properties. Pearson correlations between regional properties and disease duration in the HM group were also assessed.

**Results:**

For global topology, although both groups showed a small-world network organization, HM patients exhibited significant decreased local efficiency and clustering coefficient compared with controls. For regional topology, HM patients and controls showed highly similar hub distributions, except for three additional hub regions in HM patients including left insula, anterior cingulate and paracingulate gyri (ACG), and median cingulate and paracingulate gyri (DCG). In addition, HM patients showed significantly altered nodal betweenness centrality (BC) mainly in the bilateral inferior occipital gyrus (IOG), left superior occipital gyrus (SOG), caudate nucleus, rolandic operculum and right putamen, pallidum, and gyrus rectus compared with controls. Intriguingly, the nodal BC of left IOG was negatively correlated with disease duration in HM patients.

**Discussion:**

Our findings suggest that HM exhibited alterations in WM structural networks as indicated by decreased local specialization. This study may advance the current understanding of the pathophysiological mechanisms underlying HM.

## Introduction

High myopia (HM) is a serious health issue worldwide, characterized by visual impairment. The prevalence of HM is 2.9% globally, and up to 10–20% among young adults in East and Southeast Asia (Holden et al., 2015; Morgan et al., 2018). It is defined as the ocular refractive diopter lower than-6.00 diopters (D) or the axial length larger than 26 mm. HM is also named “pathological myopia” and “degenerative myopia,” reflecting the extensive progressive trend of pathological and degenerative changes of the neurosensory retina and choroid and may extend to the brain structure (Morgan et al., 2012; Hsu et al., 2014). Therefore, exploring the high myopia-related brain structural alterations will enhance our understanding of the pathophysiological mechanisms underlying HM.

Some previous neuroimaging studies have demonstrated changes in brain volume and white matter (WM) in HM (Huang et al., 2018; Wang et al., 2021). Besides, recent studies also demonstrated that high myopia was accompanied by neural activity changes (Ptito and Kupers, 2005) and may trigger neural plasticity (Wang et al., 2020). These studies showed the truth that HM was not merely a disease that involved the fundus structure but also closely related to the structure and function of the brain. These previous studies, however, only put the focus on the individual structural alterations and neural activities of the brain, ignoring the structural and functional communication of separated structures anatomically, which may play a crucial role in information transference and processing of the brain (van den Heuvel and Hulshoff Pol, 2010).

The diffusional kurtosis imaging (DKI) technique is a noninvasive and promising imaging modality for evaluating the microstructural characteristics of WM (Cao et al., 2013). Additionally, DKI tractography reveals the brain’s structural connectivity vividly by reconstructing the major WM tracts (Basser et al., 2000). Graph theory analysis regards the brain as a graph, in which brain regions are regarded as nodes and functional or structural connections between nodes are regarded as the edges. Through the graph theoretical analysis of the brain network, we can discover the brain characteristics at both local and global levels (Wang et al., 2016). Graph theory analysis has been increasingly prevalent in the field of neuroimaging in recent years, which provides an advanced tool for investigating the topological organization of brain networks (Wen et al., 2018). Combining the DKI technique and the graph theory analysis method allows the small-world, hub properties, and modularity in the complex brain structural network to be discovered (Fang et al., 2017).

In the present research, we constructed DKI networks to study potential abnormal mechanisms underlying brain dysfunction in HM. Briefly, we first used DKI tractography to construct individual whole-brain WM networks. Three weighted networks featured by kurtosis fractional anisotropy (KFA), fractional anisotropy (FA), and fiber number (FN) were analyzed by the graph theory method to explore the potentially altered regional and global topological properties in HM patients and if these topology alterations would correlate with the disease duration significantly. The results of our research may facilitate further recognition of the underlying neurophysiological mechanisms of HM individuals.

## Materials and methods

### Subjects

Our prospective study was approved by the medical research ethics committee of Beijing Friendship Hospital, and written informed consent was acquired from all subjects according to the Declaration of Helsinki prior to enrollment. From January 2017 to December 2019, HM patients and normal controls were recruited from outpatient clinics at our institute. Patients with the following diseases or conditions were excluded: (1) other ocular diseases, such as glaucoma, strabismus, and amblyopia; (2) unilateral high myopia; (3) psychiatric or neurological disorders; (4) brain tumor or obvious cerebral infarction; (5) any systemic diseases that may affect the brain structure, such as hypertension and diabetes; (6) MRI contraindications; (7) MR images with visible artifacts. According to the exclusion criteria, five HM patients and two NCs were excluded. A total of 30 HM patients and 33 age-and gender-matched normal controls (NCs) were enrolled in the study eventually; the uncorrected visual acuity (VA) of NCs was larger than 1.0.

### Image acquisition

MR imaging was acquired using a 3.0 T GE Discovery MR750 scanner (GE Healthcare Systems, Milwaukee, WI, United States) equipped with an eight-channel, phased-array head coil. Images are acquired using slice-interleaved encoding. DKI images were obtained using a single-shot, echo planar imaging sequence, with an encoding scheme of two *b*-values (*b* = 1,000 and 2000 s/mm^2^) along 25 diffusion-encoding directions, respectively, and *b* value of 0 s/mm^2^ along five non-diffusion-weighted images. The sequence parameters of DKI images were as follows: TR/TE = 9000/96 ms; voxel size = 1 mm × 1 mm; matrix = 256 × 256; slice thickness/spacing = 3.0/0 mm; flip angle = 90; field of view = 256 × 256 mm; frequency/phase resolution = 128 × 128; 44 axial slices. For anatomical reference and image segmentation, T1-weighted fast-spin-echo images were obtained with the following parameters: TR/TE = 8.2/3.2 ms; voxel size = 0.46875 mm × 0.46875 mm; matrix = 512 × 512; slice thickness = 1.0 mm; flip angle = 12; FOV = 240 mm × 240 mm; frequency/phase resolution = 256 × 256; 200 axial slices. Regular T2-weighted fast-spin-echo images were obtained before acquiring diffusion kurtosis images. Images with visible artifacts, obvious WM hyperintensities, cerebral infarction lesions, and brain tumors were excluded from our study.

### DKI data processing

First, we created brain masks from the b0 image to delete nonbrain tissues using the Brain Extraction Tool (BET) in FSL (v5.0, http://www.fmrib.ox.ac.uk/fsl). Second, using the eddy tool in FSL, we corrected motion artifacts and eddy current distortions of DKI images by applying an affine alignment of each diffusion-weighted image to the b0 image. Then, the Diffusional Kurtosis Estimator toolbox (v2.6, http://www.nitrc.org/projects/dke) was applied to calculate the diffusion tensor (DT) and kurtosis tensor (KT) parameters using the constrained linear least squares-quadratic programming (CLLS-QP) algorithm (Tabesh et al., 2011). The DKI model was parameterized by the DT and KT from which several rotationally invariant scalar measures were extracted. We mainly calculated the DT-derived measure including FA and the KT-derived measure including KFA.

### Network construction

We defined the network nodes (details shown in Supplementary Table S1) as our previous study (Wen et al., 2017). Each individual high-resolution structural image (T1WI image) was first coregistered to the b_0_ image in the diffusion space using a linear transformation. Then, the transformed T1 images were nonlinearly transformed to the ICBM152 T1 template in the MNI space. The resulting inverse transformations were used to warp the automated anatomical labeling (AAL) atlas (Tzourio-Mazoyer et al., 2002) from the MNI space to the individual native space, and the nearest-neighbor interpolation method was used to preserve discrete labeling values. We manually checked the quality of all registered images to remove subjects with poor registration results, and all images passed the quality check and were retained. Using this procedure, we segmented the entire cerebrum into 90 cortical and subcortical regions (45 for each hemisphere), each representing a network node.

To define the network connections, we performed diffusion tractography using a fiber tracking (FT) module embedded in DKE. The FT module uses the diffusion and kurtosis tensors from DKE for the DKI approximation of the diffusion orientation distribution function (dODF), and this module uses the closed-form solution of the kurtosis dODF derived in Jensen et al., 2014 along with the image processing algorithms developed in Glenn et al. (2015). We set the ODF optimization and tractography parameters (details shown in Supplementary materials) to perform FT using the scripts provided by the FT Module, utilizing the Euler method. Each network connection represents the connecting fiber that links two brain nodes. The weight of network connection is defined as three types: FN, FA, and KFA. Thus, for each subject, three 90 × 90 symmetric weighted networks were constructed. To remove spurious connections, each network was thresholded over a wide range of sparsity (6 to 20%, interval of 1%). The flowchart of DKI preprocessing and network construction is shown in Supplementary Figure S1.

### Network topological analysis

Graph theoretical quantitative parameters were assessed at each sparsity threshold to characterize the WM structural network topology, using a graph theoretical network analysis toolbox (*GRETNA v2.0*, http://www.nitrc.org/projects/gretna/; Wang et al., 2015). As in our previous studies (Wen et al., 2017, 2018), seven global topological measures were calculated (general descriptions for the network properties are shown in Table 1): shortest path length (L_p_), global efficiency (E_glob_), local efficiency (E_loc_), clustering coefficient (C_p_), normalized L_p_ (λ), normalized C_p_ (γ), and small-worldness (σ). For nodal measures, we considered the nodal betweenness centrality (BC) that plays a vital role in identifying hub nodes of structural networks, defined as follows:


(1) $$B_{nodal}\left(i\right)=\underset{s\neq i\neq t\in G}{\sum }\frac{e_{st}\left(i\right)}{e_{st}}$$

**Table 1:** Global and local topological properties used in the study.

	General descriptions
**Global properties**	
Shortest path length L_p_	L_p_ is defined as the average length of the shortest path between every two nodes in network G that quantifies the ability for information to be propagated in parallel, which is computed as follows: $L_{P}\left(G\right)=\frac{1}{N\left(N\_1\right)}\underset{i\neq j\in G}{\sum }L_{\mathrm{ij}}$
Global Efficiency E_glob_	E_glob_ is defined as the mean value of all regions’ global efficiency, which is computed as follows: $E_{\mathrm{glob}}\left(G\right)=\frac{1}{N\left(N\_1\right)}\underset{i\neq j\in G}{\sum }\frac{1}{L_{\mathrm{ij}}}$
Local Efficiency E_loc_	E_loc_ is defined as the mean value of all regions’ local efficiency, which is computed as follows: $E_{\mathrm{loc}}\left(G\right)=\frac{1}{N}\underset{i\in G}{\sum }E_{\mathrm{glob}}\left(G_{i}\right)$ where G_i_ denotes the subgraph composed of the nearest neighbors of node i.
Clustering coefficient C_p_	C_p_ is the average clustering coefficient over all nodes that indicates the extent of local interconnectivity or cliquishness in a network, which is computed as follows: $C_{i}\frac{2}{k_{i}\left(k_{i}\_1\right)}\underset{j,k}{\sum }{\left({\bar{w}}_{\mathrm{ij}}{\bar{w}}_{\mathrm{jk}}{\bar{w}}_{\mathrm{ki}}\right)}^{1/3}$ where k_i_ is the degree of node i, and $\bar{w}$ is the weight, which is scaled by the mean of all weights to control each subject’s cost at the same level.
Normalized L_p_ (λ)	λ = L_p_^real^/ L_p_^rand^ and L_p_^rand^ are the mean shortest path lengths of 100 matched random networks.
Normalized C_p_ (γ)	γ = C_p_^real^/ C_p_^rand^ and C_p_^rand^ are the mean clustering coefficients of 100 matched random networks.
Small-worldness σ	σ = λ/γ, A real network would be considered a small world if γ > 1 and λ ≈ 1.
**Nodal properties**	
Nodal Betweenness Centrality B_nodal_(i)	B_nodal_(i) is defined as the fraction of all shortest paths in the network G that pass through a given node i.

where e_st_(i) denotes the number of shortest paths in the network G between node s and node t, which pass through node i, while e_st_ denotes the total number of shortest paths in the network G between node s and node t. Node *i* was considered a brain hub if *B*_nodal_(*i*) was at least one standard deviation (SD) greater than the average nodal BC of the network (i.e., *B*_nodal_(*i*) > mean + SD). Notably, we calculated the average value of each topological metric under all sparsity thresholds to provide a summarized scalar independent of single threshold selection (Zhang et al., 2015). The computer specifications and Operating System for performing image processing and analysis were a CentOS 7.1 Linux system on a Huawei high-performance cluster computing platform with 12 computing nodes, 240 processor cores, and 250-TB storage capacity.

### Between-group statistical comparison and correlation analysis

A two-sample *t*-test was used to analyze the differences in the network measurements (global and nodal properties) between the two groups. Of note, the average values of nodal BC across thresholds were used to investigate brain hubs and significantly altered nodes. We also calculated the Pearson correlation between significantly altered nodal BC and disease duration in patient groups using SPSS v21.0.

## Results

### Clinical characteristics and demographic of the subjects

Thirty HM patients (age: 35.13 ± 13.73 years, range: 22–65, 19 women/11 men) and thirty-three NCs (age: 38.55 ± 18.00 years, range: 24–65 years, 19 women/14 men) were recruited in our study eventually. The baseline data of recruited subjects were well-balanced between the two groups (Table 2). No significant differences in age (*p* = 0.315, two-sample *t*-test) and gender (*p* = 0.641, chi-square test) were found between the HM and NCs.

**Table 2:** Demographic and clinical characteristics of high myopia patients and healthy controls.

Characteristic	High myopia patients	Normal controls	*p*-value
Number of subjects	30	33	
Age (years)	35.13 ± 13.73	38.55 ± 18.00	0.315^t^
Gender (female/male)	19/11	19/14	0.641^c^
Duration of illness (years)	23.8 ± 11.8	NA	
Refractive diopter_R (D)	−8.4 ± 4	NA	
Refractive diopter_L (D)	−8.5 ± 4	NA	
Axial length_R	28.0 ± 1.7	NA	
Axial length_L	27.9 ± 2.2	NA	

NA, not applicable; D, diopter; *t*, two-sample *t*-test; c, chi-square test.

### Alterations in the global properties of WM networks in HM

**Figure 1:**
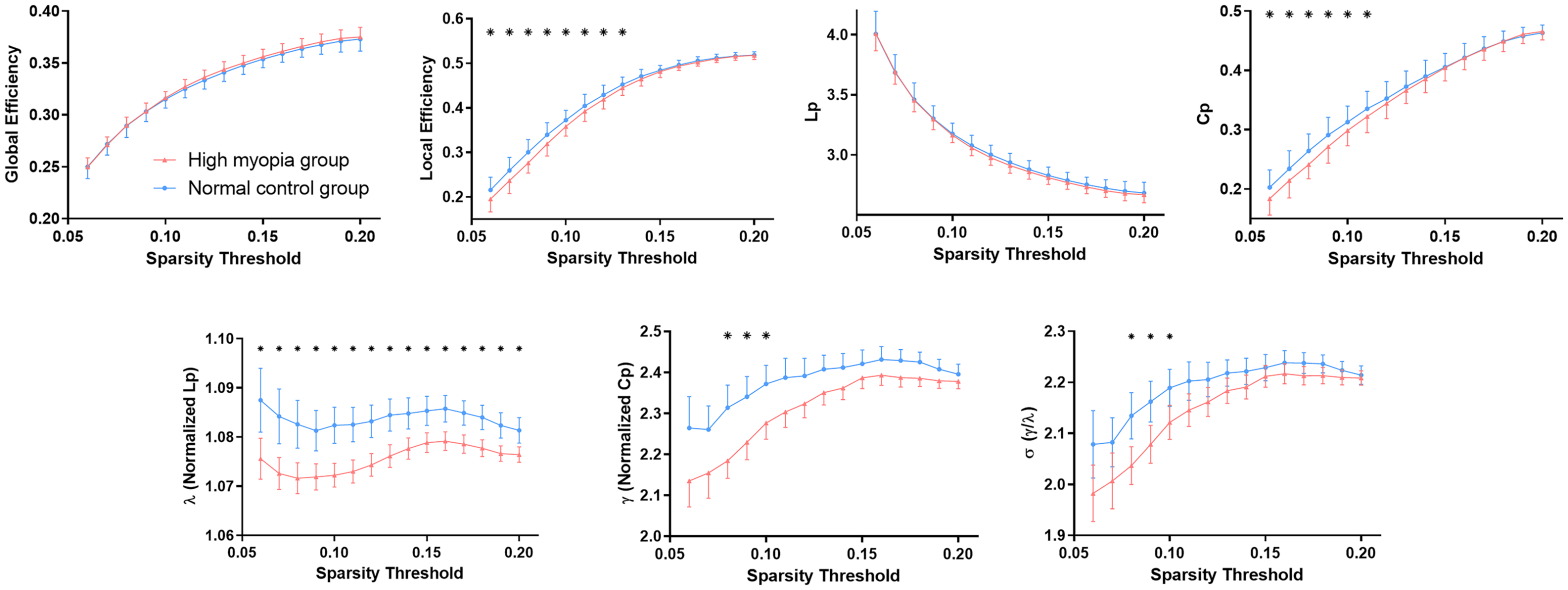
Differences in global topological properties of KFA-weighted networks between high myopia patients and controls. Data points marked with a star indicate a significant (*p* < 0.05, FDR corrected for multiple comparisons) difference between groups in the global network metric under a corresponding threshold. FDR, False discovery rate; KFA, kurtosis fractional anisotropy.

For KFA-weighted networks, both HM patients and NCs presented with a small-world property of WM networks characterized by γ > 1,λ ≈ 1 and σ = γ/λ > 1 (Cao et al., 2013; Wen et al., 2018; Figure 1). Besides, patients with HM showed significantly (*p* < 0.05) decreased C_p_, λ, γ, E_loc_, and σ over a wide range of thresholds compared with NCs. Moreover, HM subjects showed significantly decreased average values of C_p_, λ, and E_loc_ across thresholds (Table 3). Significant *p*-values were obtained after false discovery rate (FDR) correction (Benjamini and Hochberg, 1995). Nevertheless, no significant difference was found between groups in all global properties in the FA-weighted and FN-weighted networks though both groups also showed a small-world organization at each sparsity threshold (details shown in Supplementary Table S2).

**Table 3:** Group comparisons of average values of global topological metrics under all thresholds.

KFA-weighted Network	E_glob_	E_loc_	L_p_	C_p_	λ	γ	σ
NC	0.331 ± 0.009	**0.419 ± 0.013**	3.066 ± 0.087	**0.361 ± 0.018**	**1.084 ± 0.019**	2.377 ± 0.202	2.192 ± 0.156
HM	0.333 ± 0.007	**0.408 ± 0.014**	3.052 ± 0.064	**0.351 ± 0.018**	**1.075 ± 0.012**	2.308 ± 0.162	2.145 ± 0.133
*p* value	0.372	0.005^*^	0.469	0.039^*^	0.046^*^	0.152	0.222

HM: high myopia; NC: normal control.

The symbol “*” represents there have statistical differences between the two groups and the value of *p* smaller than 0.05. The bold values represent the ranges of the corresponding values.

### Alterations in the regional properties of WM networks in HM

Since significant differences between HM and NCs were only found in KFA-weighted networks, we focused on the KFA-weighted network and identified the brain structural hubs of each group based on it. A node is considered the hub region if its nodal BC is at least one SD larger than the average BC of the network (Wen et al., 2018).

**Figure 2:**
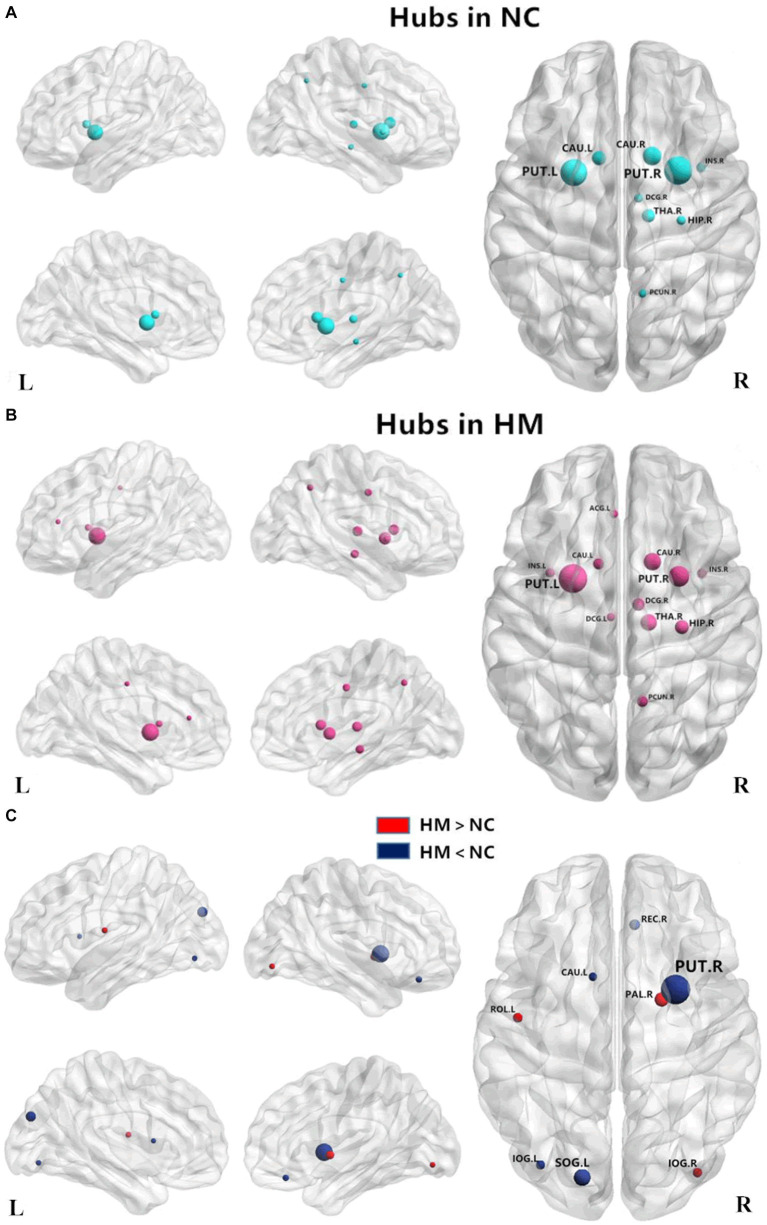
Distribution of hub regions in the WM structural networks of both groups and altered nodes in HM patients. **(A,B)** Three-dimensional representations of the hub distributions in the NC and HM groups, respectively. The hub nodes are shown in blue and red with node sizes indicating their nodal betweenness centrality values. **(C)** The disrupted nodes with significantly decreased or increased nodal betweenness centrality in the HM group are shown in blue or red, and the node sizes indicate the t values in the t-test. The brain graphs were visualized by using BrainNet Viewer software (http://www.nitrc.org/projects/bnv/). For the abbreviations of nodes, see Table 1. WM, white matter; HM, high myopia; NC, normal controls.

Patients with HM and NCs showed highly similar hub distributions, with core regions mainly in the bilateral caudate nucleus, putamen, insula, median cingulate and paracingulate gyri (DCG), left anterior cingulate and paracingulate gyri (ACG), right precuneus, thalamus, and hippocampus. Only three additional hub regions in the HM group were identified, including the left insula, ACG, and DCG (Table 4; Figures 2A,B).

**Table 4:** Hub regions of KFA-weighted networks in control and HM groups.

NC			HM		
Hub regions	Module	mean B_nodal_	**Hub regions**	Module	mean B_nodal_
INS.R	sensory/motor	23.07	INS.R	sensory/motor	21.68
DCG.R	Subcortical	21.29	DCG.R	Subcortical	25.65
HIP.R	Subcortical	21.93	HIP.R	Subcortical	28.31
PCUN.R	DMN	20.03	PCUN.R	DMN	22.68
CAU.L	Subcortical	31.21	CAU.L	Subcortical	23.19
CAU.R	Subcortical	39.80	CAU.R	Subcortical	34.66
PUT.L	Subcortical	55.30	PUT.L	Subcortical	54.93
PUT.R	Subcortical	58.06	PUT.R	Subcortical	40.59
THA.R	Subcortical	29.72	THA.R	Subcortical	33.22
			**INS.L**	sensory/motor	20.08
			**ACG.L**	DMN	18.58
			**DCG.L**	Subcortical	18.30

Bnodal represents the average value of the nodal betweenness centrality across thresholds.

**Figure 3:**
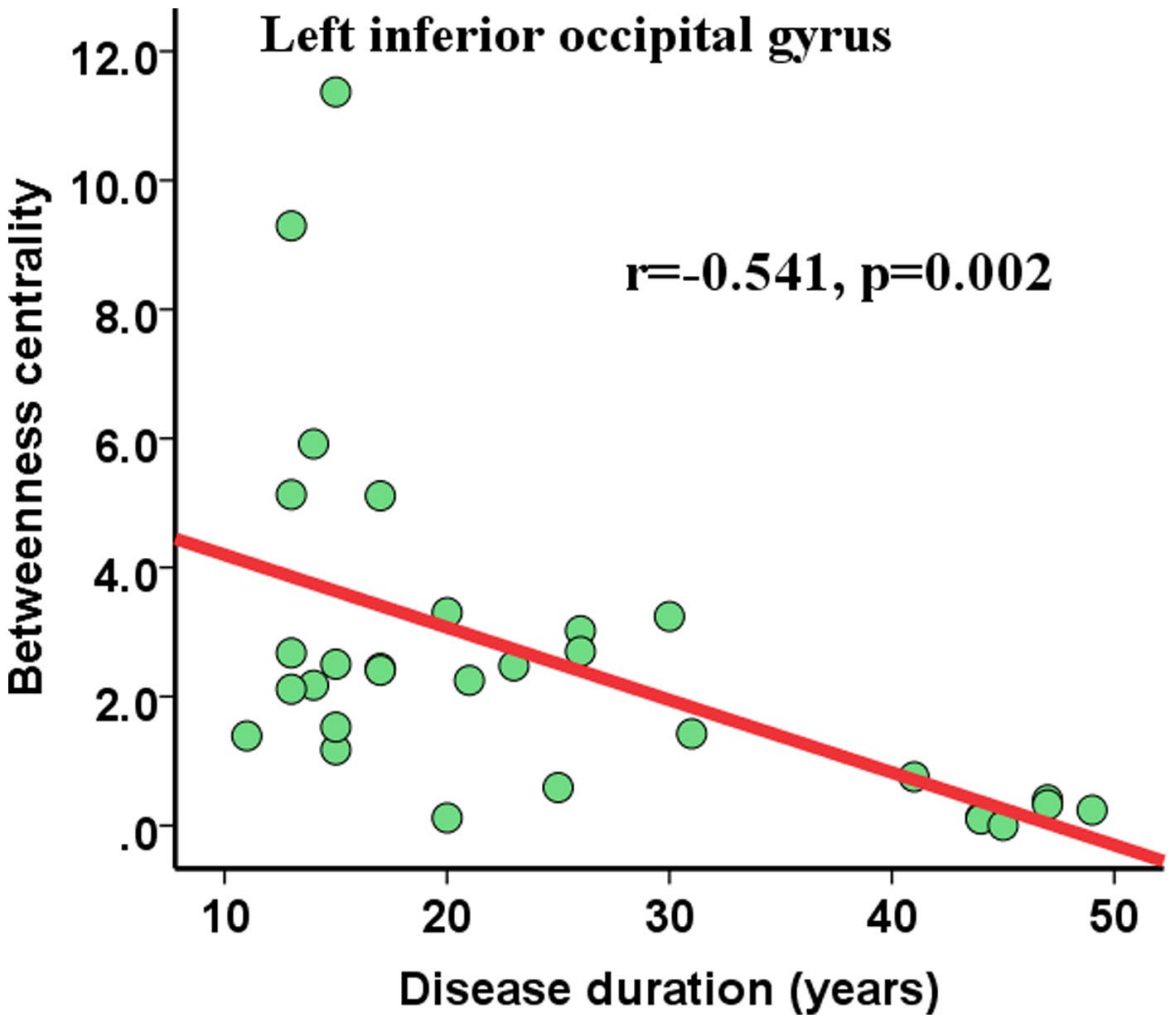
Pearson correlations between nodal topological properties and disease duration. The nodal betweenness centrality of the left inferior occipital gyrus showed significant (FDR-corrected) negative correlations with disease duration. Abbreviations: FDR, False discovery rate.

We also assessed the nodal betweenness centrality (average value across thresholds) in the two groups. Compared with NCs, patients with HM showed significantly altered nodal betweenness centrality mainly in the bilateral inferior occipital gyrus (IOG), left superior occipital gyrus (SOG), caudate nucleus, rolandic operculum and right putamen, pallidum, and gyrus rectus (Table 5; Figure 2C), involving vision, default-mode network (DMN), sensorimotor, and subcortical functional modules (Yong et al., 2009). Noteworthy, among these above-altered regions, the left caudate nucleus and the right putamen were hub regions for both groups. Pearson correlation analysis revealed a negative correlation between nodal betweenness centrality of the left inferior occipital gyrus and disease duration (*r* = −0.541, *p* = 0.002, FDR corrected for multiple comparisons; Figure 3).

**Table 5:** Brain regions showing significantly altered nodal betweenness centrality in the high myopia group for KFA-weighted networks.

		B_nodal_			
Module	Region	NC	HM	*t* value	*p* value
sensory/motor	ROL.L	2.74 ± 2.92	5.17 ± 4.70	−2.094	0.042
DMN	REC.R	5.40 ± 4.93	2.91 ± 4.00	2.182	0.031
vision	SOG.L	13.13 ± 12.31	5.97 ± 6.83	2.570	0.031
vision	IOG.L	3.67 ± 4.19	1.83 ± 2.59	2.064	0.043
vision	IOG.R	0.82 ± 1.14	1.94 ± 2.72	−2.085	0.044
Subcortical	CAU.L	31.36 ± 17.92	23.19 ± 13.66	2.017	0.048
Subcortical	PUT.R	58.06 ± 25.56	40.59 ± 13.84	3.413	0.001
Subcortical	PAL.R	5.43 ± 4.54	9.06 ± 7.26	−2.395	0.020

Bnodal represents the average values (mean ± SD) of the nodal betweenness centrality of each group.

## Discussion

In this study, we reconstructed the individual whole-brain, ROI-level WM networks and then performed graph theory analysis to explore the regional and global network topological properties in HM subjects and normal controls. The major findings of our study were as follows: (1) Both HM and NCs presented with an efficient small-world organization in all types of weight networks, (2) Characterized by KFA-weighted networks, HM patients showed significantly decreased Eloc and C_p_ in comparison to NCs, (3) Despite nine common hub distributions in both groups, three more hubs (left insula, ACG, and DCG) appeared in the HM patients compared with NCs, (4) HM patients showed widespread regional topological changes characterized by nodal BC, mainly involving brain regions within vision, DMN, sensorimotor, and subcortical functional modules, and (5) Significantly negative correlations between the BC values of the left inferior occipital gyrus with disease duration in HM were found.

### Topological property comparison in different networks

In this study, three types of networks (FN, FA, and KFA-weighted network) were constructed. However, topological attribute alterations between the patients with HM and NCs were only found in KFA-weighted networks rather than FN or FA-weighted network. It was suggested that the KFA-weighted network dried from the DKI model was more sensitive in reflecting the topological attributes in WM structural network than the FA network dried from the DTI model. The DTI model is based on the hypothesis that water molecules were in Gaussian distribution in the biological tissue (Veraart et al., 2011). However, the displacement distribution of water molecules often manifests as non-Gaussian distribution. As the extension of the DTI, DKI well reflected the non-Gaussian diffusion pattern of water molecules in the tissue by evaluating the excess kurtosis of the displacement distribution (Veraart et al., 2011; Zhu et al., 2015). Conclusively, as verified in our study, DKI had a better sensitivity and practicality in identifying the pathological and developmental changes in the brain as compared to DTI.

### Small-world topology of the WM networks alterations in HM

Brain networks with small-worldness properties were found in both HM and NCs. Small-worldness network has efficient information transmission and processing capabilities (Wang et al., 2016)， which has been proven by various modal images, such as MRI, magnetoencephalography, and electroencephalography (Stam, 2004; Zhu et al., 2017; Yang et al., 2019). Small-world property enables the brain network’s shortest path lengths and high clustering coefficients and thus achieves the best balance between global integration and local specialization (Shu et al., 2009). Our results agreed with previous research and support the view that a small-worldness network can accommodate developmental alteration and disease conditions (Cao et al., 2013).

Despite the common small-world topological properties, HM subjects showed decreased E_loc_ and C_p_. However, it should be noted that no significant difference in the glob efficiency and shortest path was identified between the two groups. The E_loc_ and E_glob_ represent the information transmission efficiency of the local and glob brain network, respectively. The C_p_ reflects the degree of clustering between nodes and the modularity of the network. The shortest path was the most efficient path for the information transmission between nodes in the network, with maximum efficiency and the least energy consumption (Jiang et al., 2021). These results in our present study showed that the destruction of the brain network in HM mainly involved the efficiency of local information transmission. The overall information transmission efficiency of the brain and the shortest path for information transmission were not affected. In the previous study on DKI, the tract-based spatial statistics method was used to analyze brain microstructural abnormalities in HM (Wang et al., 2021), and it was found that HM subjects presented microstructural damage in motor conduction and vision-related brain regions. We assumed that the reduction of the ability to handle local information in HM may be attributable to the microstructural damage in WM.

One diffusion tensor tractography study analyzed the global topological properties in blind people and reported that the adolescent-blind and late-blind had significantly reduced C_p_ and E_loc_, but comparable E_glob_ and L_p_ to that of the sighted controls (Li et al., 2013). Besides, decreased E_glob_ was observed in the congenitally blind and early blind compared with the sighted controls (Li et al., 2013). It suggested that late visual defects after the completion of neurodevelopment may only result in a reduction in local efficiency but cannot affect global efficiency. For the onset ages of HM patients in our study, after establishing an intact and efficient anatomical network in the human brain, our results were consistent with this previous study. Given the fact that the small-worldness property represents the best balance between local specialization and global integration (Cao et al., 2013), our findings may be a reminder that the brain always keeps in a dynamic process of development, plasticity, and disuse to reach an optimal balance in a certain period.

### Hub-distributing reorganization and regional topology changes in HM

Hub regions of the WM networks for each group based on KFA-weighted networks were identified. We found the hub distributions in both groups showed high similarity. The common core regions were mainly in the bilateral caudate nucleus, putamen, insula, median cingulate and DCG, left ACG, right precuneus, thalamus, and hippocampus. However, there were three additional hub regions including the left insula, ACG, and DCG in HM. Insula is a part of the multisensory integration center for spatial orientation and eye movement (Yin et al., 2021). The cingulate gyrus belongs to the limbic system and is involved in multiple functions, such as memory, attention, and cognitive function. Additionally, ACG has been shown to be associated with cognitive-related visual functions, participating in the recognition and classification of visual objects (Zhang et al., 2021). The neural activity alterations of the insula (Chen et al., 2019) and cingulate gyrus (Samanchi et al., 2021) have been reported in other visual-related diseases. The three more hubs in HM subjects suggested that long-term myopia is accompanied by hub-distributing reorganization, with the function of information transference and processing of the vision, memory, and cognitive and attention enhancement. The three core nodes were all located on the left side, and we speculated that it may be related to the dominant hemisphere.

The regional topology alterations between the two groups were also analyzed, and we observed that the HM group had widespread altered nodal BC, involving areas within vision-related (left SOG and bilateral IOG), subcortical (left caudate nucleus, right putamen, and right pallidus), sensorimotor (left rolandic operculum), and default mode network (right gyrus rectus) functional modules. It should be noted that the caudate and putamen were core nodes, which manifested BC value decreased in HM compared to NCs. The Caudate and putamen are parts of the dorsal striatum and are vital to eye movement (Yin et al., 2021) and visually-guided decision-making (Moss et al., 2021). Structural alterations in the caudate and putamen were reported in other ophthalmic diseases (Shu et al., 2009; Zikou et al., 2012). Although no study has demonstrated structural changes in the dorsal striatum in myopia subjects, we speculated patients with HM might have functional weakness of the eye movement combined with impaired visually guided decision-making based on our results, and long-term visual deficit may incorporate structural plasticity and hub distributing reorganization of the brain in HM.

### Correlations between nodal betweenness centrality and disease duration

The relationships between the nodal BC values and the disease duration in HM were analyzed. It was demonstrated that the BC value of left IOG negatively correlated with disease durations. The occipital lobe is mainly responsible for visual information processing, and the IOG plays a crucial role in face processing (Sato et al., 2017). The hypofunction of the left IOG and its negative correlation with disease duration reflected the visual function gradually damaging and weakening in our study. Consistently, Li et al. (2013) found a significant correlation between the ages of blindness onset and the topological properties of anatomical networks in blind individuals. These findings suggested that abnormal visual information input can cause abnormality in the brain structure and induce neural reorganization in the brain.

## Conclusion

In summary, our preliminary results demonstrated that HM incorporates structural plasticity and reorganization of the brain, mainly manifesting as local specialization decrease in WM structural networks. DKI based on the graph theoretical analysis is a promising and novel approach for detecting topological properties of brain networks in HM, which provides new perspectives on the understanding of the brain’s re-organization and pathophysiological mechanisms in the specific population with high myopia.

## Data availability statement

The data analyzed in this study is subject to the following licenses/restrictions: The datasets generated for this study are available on request to the corresponding author. Requests to access these datasets should be directed to cjr.wzhch@vip.163.com.

## Ethics statement

The studies involving human participants were reviewed and approved by The medical research ethics committee of Beijing Friendship hospital. The patients/participants provided their written informed consent to participate in this study.

## Author contributions

HHW: conceptualization, data curation, investigation, methodology, formal analysis, resources, and writing - original draft. HWW: data curation, investigation, methodology, formal analysis, and software. JL: conceptualization and data curation. QC: formal analysis and software. SSL: data curation and supervision. ZCW: writing - review and editing, supervision, validation, project administration, and funding acquisition. All authors contributed to the article and approved the submitted version.

## Funding

This work was supported by the National Natural Science Foundation of China (grant numbers: 61527807, 32100902, 81800840, 62171297, 81701644, and 61801311), the Beijing Scholars Program [grant number: (2015) 160], the Beijing Municipal Administration of Hospitals (grant numbers: SML20150101, PX2018001, YYZZ2017A14, and YYZZ2017B01), the Beijing Chao-Yang Hospital Golden Seeds Foundation (CYJZ202156), and the Fundamental Research Funds for the Central Universities (SWU118065).

## Conflict of interest

The authors declare that the research was conducted in the absence of any commercial or financial relationships that could be construed as a potential conflict of interest.

## Publisher’s note

All claims expressed in this article are solely those of the authors and do not necessarily represent those of their affiliated organizations, or those of the publisher, the editors and the reviewers. Any product that may be evaluated in this article, or claim that may be made by its manufacturer, is not guaranteed or endorsed by the publisher.
