# Supplementary material for: Disrupted topological organization of white matter structural networks in high myopia patients revealed by diffusion kurtosis imaging and tractography
Source: Front Neurosci. 2023 Jun 22;17:1158928. doi: 10.3389/fnins.2023.1158928 (PMC10324656; doi:10.3389/fnins.2023.1158928)
Supplement: Supplementary file 1 [file Data_Sheet_1.docx]

**Supplementary materials**

**Materials and methods**

**Network construction**

We set the ODF optimization and tractography parameters to perform white matter fiber tracking (FT) using the scripts (ft_parameters.txt) provided by the FT Module, and the detailed parameters were as follows:

%--------------------------------------------------------------------------

%USER OPTIONS

%--------------------------------------------------------------------------

studydir = '***/path to data/***'; %Full path to directory containing DT.mat, KT.mat, and fa.nii files.

subject_list={''}; %Specify subject numbers (i.e. {'Subject01','Subject02','Subject03',...}).

%ODF Optimizatinon

odf_optimization = 1; %Analyze the kurtosis dODF (1) or skip odf optimization (0). This step must be performed prior to tractography.

sd = 4; %[3,4, or 5] Sampling distribution to use. See sphericalgrid3(4,5).m documentation for more info.

quasiNewton = 1; %Apply non-linear optimization to increase accuracy of peak detection (1) or use orientations directly from the pre-defined sampling distribution (0).

radial_weight = 4; %Radial weighting power.Recommended radial_weight = 4.

release_memory = 0; %Do not release memory (0), release memory after each subject (1), or release memory more frequently (2). See User's Guide for additional info.

wrt_flg = 1; %Write outputs (1) or don't write outputs (0).

pre_name = ''; %String to append to the beginning of output names.

post_name = ''; %String to append to the end of output names.

%Tractography

tractography_flg = 1; %Perform tractography (1) or don't perform tractography (0).

fa_threshold = 0.1; %FA threshold.

angle_threshold = 35; %Angle threshold in degrees.

trk_length = 20; %Minimum tract length in mm.

step_size = 1; %Step size in mm (0 defaults to half of the voxel length).

trk_mask = ''; %Path to tracking mask to apply in addition to other tract termination criteria defined above. Should be in the same orientation as the fa.nii image.

seednum = 1E5; %Number of random seed points in the tracking mask.

shift = 0.5; %Shift applied to voxel coordinates in .trk file.

output_DTI_trks = 1; %Include tractography performed from just the diffusion tensor (1) or don't (0).

image_orientation = 'LAS'; %Orientation of input image volumes. By convention, spm writes images in 'LAS.'

odf_orientation = 'LAS'; %Orientation of the gradient table used to estimate the diffusion and kurtosis tensors. If SPM is used to estimate the gradient tables, this should be 'LAS.'

**Supplementary Table S1**. The 90 cortical and subcortical regions of interest defined in our study.

| Regions | Abbr. | Regions | Abbr. |
| --- | --- | --- | --- |
| Precentral gyrus | PreCG | Lingual gyrus | LING |
| Superior frontal gyrus, dorsolateral | SFGdor | Superior occipital gyrus | SOG |
| Superior frontal gyrus, orbital part | ORBsup | Middle occipital gyrus | MOG |
| Middle frontal gyrus | MFG | Inferior occipital gyrus | IOG |
| Middle frontal gyrus orbital part | ORBmid | Fusiform gyrus | FFG |
| Inferior frontal gyrus, opercular part | IFGoperc | Postcentral gyrus | PoCG |
| Inferior frontal gyrus, triangular part | IFGtriang | Superior parietal gyrus | SPG |
| Inferior frontal gyrus, orbital part | ORBinf | Inferior parietal, but supramarginal and angular gyri | IPL |
| Rolandic operculum | ROL | Supramarginal gyrus | SMG |
| Supplementary motor area | SMA | Angular gyrus | ANG |
| Olfactory cortex | OLF | Precuneus | PCUN |
| Superior frontal gyrus, medial | SFGmed | Paracentral lobule | PCL |
| Superior frontal gyrus, medial orbital | ORBsupmed | Caudate nucleus | CAU |
| Gyrus rectus | REC | Lenticular nucleus, putamen | PUT |
| Insula | INS | Lenticular nucleus, pallidum | PAL |
| Anterior cingulate and paracingulate gyri | ACG | Thalamus | THA |
| Median cingulate and paracingulate gyri | DCG | Heschl gyrus | HES |
| Posterior cingulate gyrus | PCG | Superior temporal gyrus | STG |
| Hippocampus | HIP | Temporal pole: superior temporal gyrus | TPOsup |
| Parahippocampal gyrus | PHG | Middle temporal gyrus | MTG |
| Amygdala | AMYG | Temporal pole: middle temporal gyrus | TPOmid |
| Calcarine fissure and surrounding cortex | CAL | Inferior temporal gyrus | ITG |
| Cuneus | CUN |  |  |


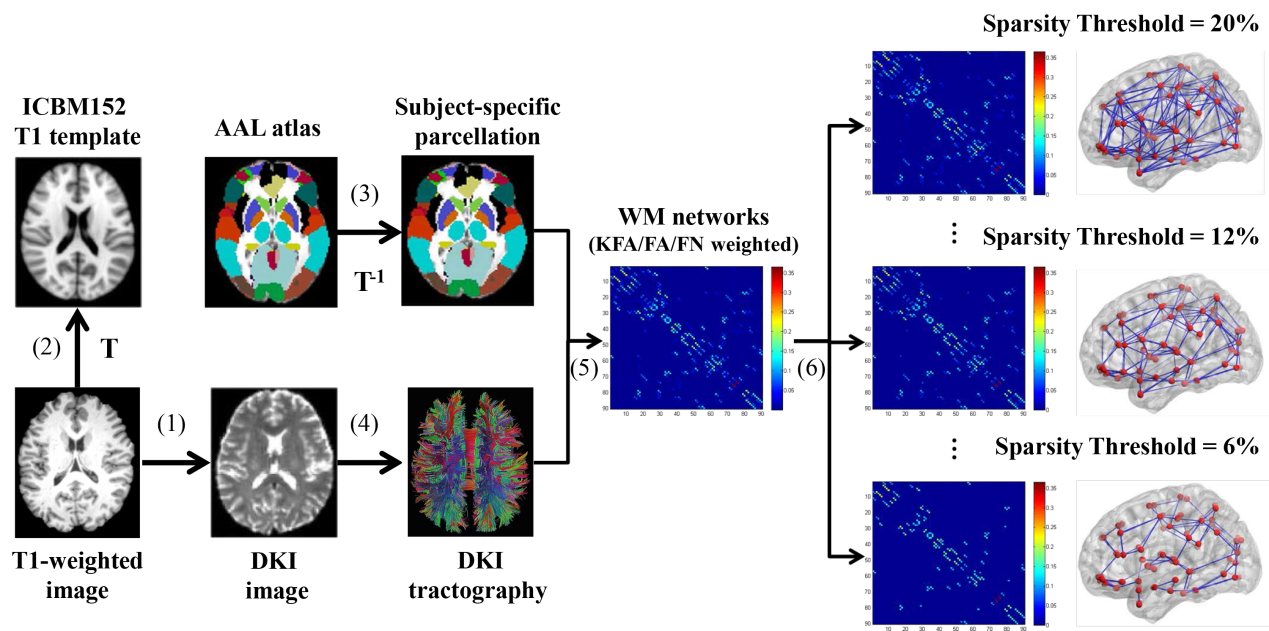


**Figure S1.** Flow chart of DKI preprocessing and network construction. (1) The T1-weighted image of each subject was first coregistered into DKI native space using rigid transformation to the b0 image. (2) The resultant T1 image was then non-linearly registered to the ICBM 152 template in the MNI space to obtain transformation matrix T. (3) The AAL atlas was then inversely warped back to the individual DKI space using the inverse transformation (T^−1^), gaining a subject-specific parcellation of node regions in native space. (4) Whole brain white matter fibers were reconstructed using a deterministic tractography method in native DKI space using DKE toolbox and fiber tracking module. (5) The WM fibers connecting any pair of regions were located and three types of edge weight between the two regions were calculated from the fiber number (FN) of the two cortical regions, and corresponding averaged KFA and FA values along WM fibers. (6) Threshold the individual matrix using a sparsity ranging from 6% to 20% with an interval of 1%, for subsequent graph theory analysis.

**Results**

**Alterations in the Global Properties of WM Networks in HM**

**Supplementary** Table S2. Group comparisons of average values of global topological metrics under all thresholds for FA-weighed and FN-weighted networks.

| FA-weighted  Network | E_glob_ | E_loc_ | L_p_ | C_p_ | λ | γ | σ |
| --- | --- | --- | --- | --- | --- | --- | --- |
| NC | 0.154±0.004 | 0.178±0.008 | 6.586±0.197 | 0.274±0.026 | 1.075±0.018 | 2.047±0.222 | 1.902±0.181 |
| HM | 0.154±0.004 | 0.178±0.006 | 6.567±0.183 | 0.273±0.019 | 1.069±0.013 | 2.079±0.171 | 1.942±0.142 |
| p value | 0.701 | 0.896 | 0.685 | 0.830 | 0.187 | 0.525 | 0.337 |
| FN-weighted  Network | E_glob_ | E_loc_ | L_p_ | C_p_ | λ | γ | σ |
| NC | 8.617±1.017 | 14.442±1.525 | 0.117±0.013 | 0.043±0.009 | 1.215±0.066 | 3.549±0.245 | 2.929±0.187 |
| HM | 8.547±1.096 | 14.100±1.793 | 0.119±0.017 | 0.043±0.009 | 1.208±0.054 | 3.565±0.172 | 2.957±0.165 |
| p value | 0.792 | 0.416 | 0.702 | 0.975 | 0.663 | 0.739 | 0.529 |

HM: high myopia, NC: normal control, FN: number of fibers.
